# Physiological mechanism of action and partial separation of herbicide–active compounds from the *Diaporthe* sp. extract on *Amaranthus tricolor* L.

**DOI:** 10.1038/s41598-023-46201-0

**Published:** 2023-10-31

**Authors:** Nutcha Manichart, Chamroon Laosinwattana, Naphat Somala, Montinee Teerarak, Nawasit Chotsaeng

**Affiliations:** 1 Department of Plant Production Technology, School of Agricultural Technology, King Mongkut’s Institute of Technology Ladkrabang, Bangkok, 10520 Thailand; 2 Department of Chemistry, School of Science, King Mongkut’s Institute of Technology Ladkrabang, Bangkok, 10520 Thailand; 3 Advanced Pure and Applied Chemistry Research Unit (APAC), School of Science, King Mongkut’s Institute of Technology Ladkrabang, Bangkok, 10520 Thailand

**Keywords:** Microbiology, Physiology

## Abstract

Thirteen fungi that produce compounds with herbicidal activities were isolated, identified, and extracted under the assumption that the mechanism of action occurs during seed exposure to the extract. The extracts from all the fungal strains considerably decreased the growth parameters of *Amaranthus tricolor* L. The EC010 strain extracts showed the greatest effect. Through ITS region gene sequencing methods, the isolated EC010 was identified as a genus of *Diaporthe*. The results showed a significant (*p* < 0.05) inhibitory effect of 91.25% on germination and a decrease in shoot and root length by 91.28% and 95.30%, respectively. The mycelium of *Diaporthe* sp. was extracted using sequential extraction techniques for the partial separation of the herbicidal fraction. According to the bioassay activities, the EtOAc fraction showed the highest inhibitory activity. The osmotic stress of the *A. tricolor* seeds was studied. Although the extract increased the accumulation of proline and soluble protein, the treated seeds showed lower imbibition. While the activity of α-amylase was dramatically decreased after treatment. A cytogenetic assay in the treated *Allium cepa* L. root revealed a decrease in the mitotic index, an altered mitotic phase index, and a promotion of mitotic abnormalities. Accordingly, the *Diaporthe* sp. may serve as a potential herbicidal compound resource.

## Introduction

Globally, nearly 3 billion kg of pesticides are used every year with a budget of roughly 40 billion USD^1^. According to the Thailand Ministry of Agriculture and Cooperatives, in 2021, approximately 136,140 tons of agrochemical substances were imported. Herbicides were the largest importation (54.51%), followed by insecticides (21.71%) and fungicides (17.81%). Although herbicides seem an indispensable part of the agricultural process, global interest in the indiscriminate use of agrochemicals on human health is a public concern^2^. Consequently, many countries around the world have regulation policies in place to restrict and ban some herbicides today, including paraquat and glyphosate^3^. It is prohibited in over 50 countries due to its high toxicity and association with suicide^4,5^. Since 2007, the European Union has enforced a ban on paraquat, and in the USA, it is restricted to licensed applicators only^6^. In 2015, the International Agency for Research on Cancer categorized glyphosate as a human carcinogenic risk^3,6,7^. Conversely, in 2018, the U.S. National Cancer Institute did not establish a link between glyphosate-based herbicides and cancer^8^. Consequently, pesticide regulators, notably the U.S. Environmental Protection Agency and the European Food Safety Authority, maintain that the use of glyphosate is considered acceptable when used as labelled^3^. However, several Asian governments, such as those in Thailand, Vietnam, the Philippines, and Indonesia, still have established restrictions on glyphosate use in agriculture^3,9^. The current situation provides a need and opportunity to find alternative methods for weed management.

Natural herbicides are products that naturally originate from metabolites of living organisms, and they are used to control weed populations without harming the environment^10,11^. There is a need for eco-friendly and rapidly degrading natural products, but only a few microorganisms and one plant (9 fungi, 3 bacteria, and 1 plant extract) products are commercially available in the current markets^12^, including triketones, cinmethylin, bialaphos, and glufosinate. Therefore, searching and finding the sources of metabolites with herbicidal activities is an important and necessary tool to produce natural herbicides. Fungi are used as a source of commercially exploitable products^13^ because they produce several biomolecules with different structural and biological characteristics to compete with or defend from surrounding species. The release of chemicals into the environment by one species to affect another species is called allelopathy^14^. The evidence for allelopathic interactions and the potential of their compounds based on the evaluation of seed germination and seedling growth of some plants was published^15–17^. The genera *Alternaria, Fusarium*^15^, *Colletotrichum*^18^, *Curvularia*, *Diaporthe*^13^, *Myrothecium*^19^, *Phoma*^20^, *Phomopsis*, *Puccinia*, *Pythium*, and *Trichoderma*^21^ have been recorded as natural herbicidal agents that inhibit seed germination and the growth of weeds. The allelochemicals are absorbed by the weed seeds, initiating damage to the cell membrane, DNA, mitosis, and amylase activity, altering the hormone balance and other biochemical processes, and delaying or inhibiting seed germination^22^.

The goal of the present study was to isolate phytopathogenic fungi from weed symptoms to produce biomolecules. We aimed to find a source of herbicide-active compounds and determine the mode of action by which the extract carries out these allelopathic effects. Thirteen fungi were isolated, and we extracted the biomolecules from their mycelia. The extracts were applied to an amaranth (*Amaranthus tricolor* L.) test plant. The selection of fungi was based on the inhibition of pre-emergence bioassay results. We hypothesized that this would occur by interfering with (i) osmotic stress, (ii) the energy provided for the germination process, and (iii) mitosis of the meristematic cell. We also investigated the physiological changes in the seeds that occurred during exposure to the extracts. The strain with the greatest potential was identified through molecular analyses.

## Results and discussion

### Screening of the herbicidal strain

In this study, thirteen phytopathogenic fungus strains from the eight weeds were isolated from Ladkrabang, Bangkok, Thailand, and SMF was used to produce fungal mycelium. All the fungal strains exhibited characteristic colonies and morphologies that could be used to differentiate the isolates. The highest number of phytopathogenic fungi (3 strains) in this study was isolated from the plant *Phyllanthus niruri* L. (Table 1). The percentage yield of 75% (v/v) EtOH extract ranged from 1.13 to 6.20%. Table 2 shows all the fungal extracts and their inhibitory effect on seed germination and the early growth of weeds at different levels as part of the primary screening. Regarding seed germination, the most pronounced effect was observed for fungi EC010 and EC005-2. Other fungi also showed an herbicidal effect, but these were at a level lower than 30.00% over the control. The data indicated that shoot and root growth were inhibited by all the tested fungi, and root length was inhibited to a greater magnitude than shoot length. However, extracts of the EC010 and EC005-2 isolates also showed the highest inhibition levels. The EC010 and EC005-2 crude extracts were diluted to a lower concentration. The results are shown in Table 3. The crude extract of both strains dose-dependently decreased the germination percentage and early growth. The degree of inhibition increased as the extract concentration increased. Overall, the phytotoxic effects of these extracts on seedling growth were similar to those observed for the germination parameter.

**Table 1:** Weed species collected, and number of phytopathogenic fungi isolated in each plant and yields (% over mycelia dried weigh; DW) of the ethanol crude extract.

Collected plant species	Family	Number of fungi isolated	Strain name (code)	Mycelia yields (g/L; mycelia DW/medium)	Crude yields (% Over mycelia DW)
*Phyllanthus niruri* L	Euphorbiaceae	3	PN 001-1	2.50	1.13
			PN 001-2	3.06	2.00
			PN 001-3	2.40	4.07
*Dactyloctenium aegyptium* Willd	Poaceae	2	DA 002-1	2.80	6.20
			DA 002-2	4.55	5.82
*Eichhornia crassipes* (Mart.) Solms	Pontederiaceae	2	EC 005-1	4.45	3.93
			EC 005-2	5.00	4.00
*Ipomoea aquatica* Forssk	Convolvulaceae	1	IA 008	2.08	4.50
*Echinochloa crussgalli* (L.) Beauv	Poaceae	1	EC 010	4.75	5.11
*Mimosa pudica* L	Leguminosae	1	MP 011-2	4.78	3.21
*Gomphrena celosioides* Mart	Amaranthaceae	2	GC 012-1	2.66	4.32
			GC 012-2	3.08	4.00
*Tridax procumbens* L	Compositae	1	TP 013	1.78	2.80

**Table 2:** Inhibitory effects of 1.5 mg/mL of an ethanolic extract from thirteen isolated fungi on *A. tricolor* seed germination and early growth after 7 days of treatment.

Strain	Inhibition level (% over control)		
	Seed germination	Shoot length	Root length
PN 001-1	17.50 cde	9.88 f	52.51 de
PN 001-2	28.75 cd	24.42 def	37.37 g
PN 001-3	25.00 cd	16.67 def	47.29 ef
DA 002-1	28.75 cd	27.33 de	60.33 cd
DA 002-2	30.00 c	20.54 def	59.29 cd
EC 005-1	18.75 cde	29.26 d	61.38 cd
EC 005-2	63.75 b	64.15 b	91.13 a
IA 008	15.00 cde	27.33 de	42.59 fg
EC 010	91.25 a	91.28 a	95.30 a
MP 011-2	11.25 cde	50.58 bc	58.77 cd
GC 012-1	22.50 cd	45.74 c	56.16 de
GC 012-2	28.75 cd	44.77 c	65.55 c
TP 013	10.00 de	46.71 c	78.60 b
Surfactant	5.00 e	13.18 ef	8.35 h

The data are presented as mean. Different letters in the same column indicated significant differences according to Tukey’s multiple range tests at *p* < 0.05 level.

**Table 3:** Growth response of amaranth after exposure to different concentrations of crude ethanolic EC 005-2 and EC 010 extract.

Strain (concentration; mg/mL)	Inhibition level (% over control)		
	Seed germination	Shoot length	Root length
EC 005-2 strain			
1.50 mg/mL	63.75 b	64.15 b	91.13 ab
0.75 mg/mL	50.00 c	43.80 c	64.51 d
0.375 mg/mL	20.00 e	7.95 d	41.54 e
EC 010 strain			
1.50 mg/mL	91.25 a	91.28 a	95.30 a
0.75 mg/mL	88.75 a	74.81 b	87.47 b
0.375 mg/mL	33.75 d	45.74 c	73.38 c
Surfactant	5.00 f	13.18 d	8.35 f

The data are presented as mean. Different letters in the same column indicated significant differences according to Tukey’s multiple range tests at *p* < 0.05 level.

Isolating and purifying compounds produced by pathogens from weeds is a very feasible approach for exploiting micro-herbicides. The microbes have been reported as natural herbicides for weed control, such as anisomycin, the secondary metabolite produced by *Curvularia* sp. Q2-200^23^. It has a high herbicidal activity on *Digitaria sanguinalis* and *E. crusgalli*. Bilanaphos, obtained from *Streptomyces*, and is a lead compound of methoxyphenone and glufosinate^24^. The activity of the fungal crude extract was due to its complex mixture of various allelochemicals that contributed to its herbicidal properties. We found that the EC010 crude extract had the highest activity. The crude yield from EC010 was 5.11% based on dry mycelia (Table 1). Although all the fungal strains showed a reduction in seed germination, we observed differences in the degree of inhibition (Table 2). These differences depended on the major chemical composition and the allelopathic effects of each fungus species.

The limitations of living microbe pesticides, such as their narrow control spectrum, short shelf life, and demanding environmental requirements, can be overcome using allelochemicals from microorganisms instead^25^. Most fungi produce toxins during spore production, such as *Alternaria alternata* and *Botrytis cinerea*^26^. In this study, no sporangium or oospores were found when the isolated EC010 was cultured, which indicated that mycelium played an important role during the production of herbicidal compounds and that the mycelium extracts strongly inhibited the growth of the weed. The isolated EC010 extract was the most effective in inhibiting amaranth (91.25%) germination (Table 3). Shoot and root lengths were also inhibited by the extract of this fungus. Considering the pre-emergence evaluation, the isolated EC010 strain presented the highest herbicidal activity of the target species. Consequently, this sample was selected for further testing.

### Molecular identification of isolated EC010

**Figure 1:**
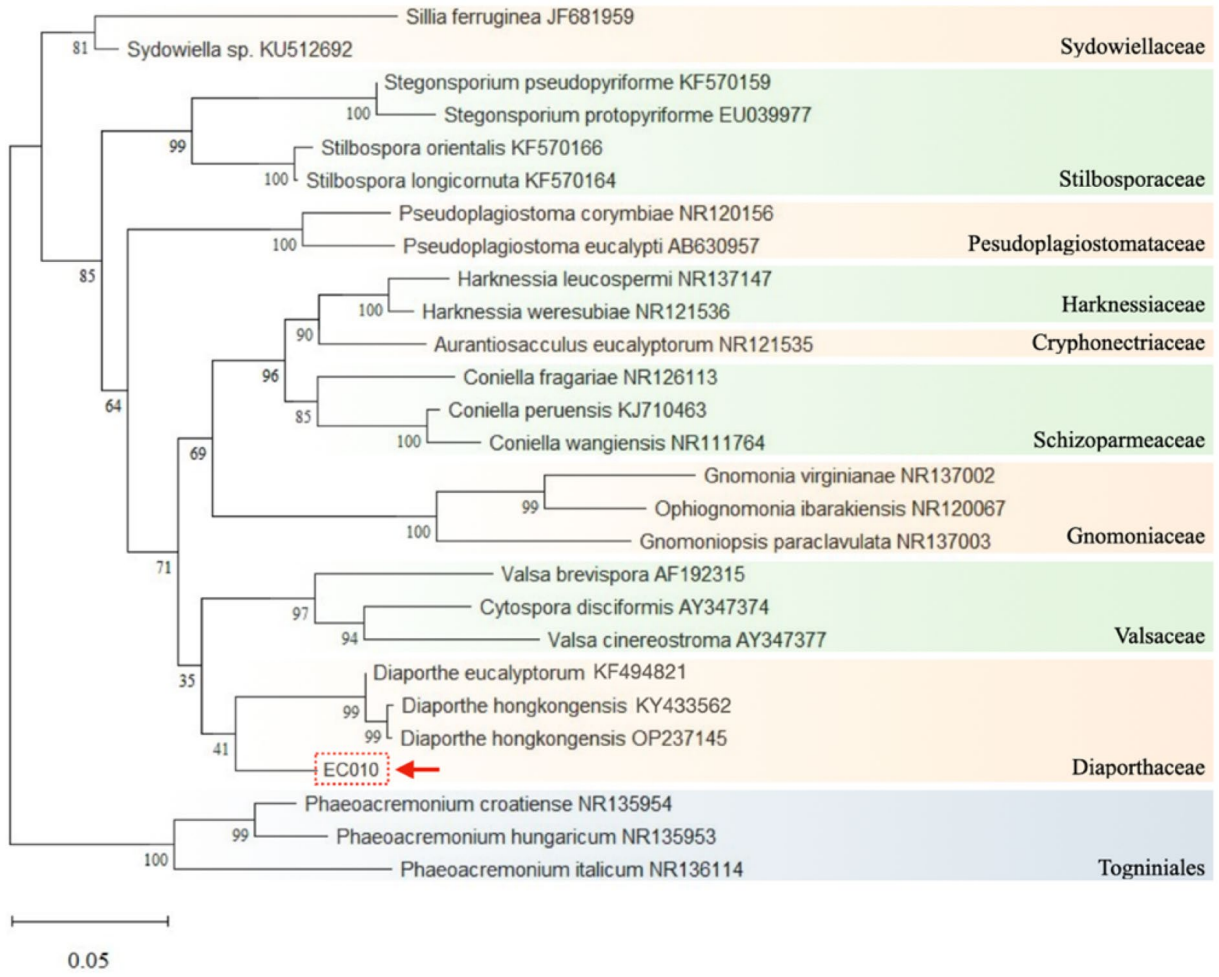
Phylogenetic tree of the EC010 isolate. This phylogenetic tree was analyzed using the ITS1 and ITS 4 regions and inferred 23 taxa under Order Diaporthales. The EC010 sequenced species is shown with a red arrow. The numerical values at the nodes indicate the Bootstrap support.

Treatments with the isolated EC010 caused the most noticeable phytotoxicities against *A. tricolor*. The fungal DNA was extracted to identify the microorganism. PCR amplification and sequencing of the ITS1 and ITS4 regions of fungal ribosomal DNA (rDNA) revealed a 498-bp nucleotide sequence deposited in NCBI, accession numbers OR143425. On the NCBI-BLASTn (National Center of Biotechnology Information) analysis, the sequence displayed the highest pairwise similarity (94.38%) with *Diaporthe hongkongensis* and *D. eucalyptorum* (accession numbers KY433562 and KF494821, respectively). A phylogenetic tree was constructed based on the ITS sequence of the isolate and its nearest relatives under the Diaporthales (Fig. 1). The Maximum Likelihood tree showed that the isolated EC010 strains clustered into the clade Diaporthaceae, with a bootstrap confidence value of 41%. Based on these two methods, these results suggest that the EC010 isolate belongs to the genus *Diaporthe*.

The molecular analysis of isolated EC010 showed a high similarity to the species *D. hongkongensis* and *D. eucalyptorum* among the nucleotide sequences available in the NCBI database. No significant differences among these species were found to identify the EC010 isolate at the species level. Moreover, the phylogenetic clade formed (Fig. 1) did not support species separation because of the low bootstrap values. According to Santos et al.^27^ and Souza et al.^13^, to achieve a sufficient delineation of the species within the *Diaporthe* genus, the classification based on morphology, mating type, and DNA sequence requires refinement. The genus *Diaporthe* spp. (teleomorph of the genus *Phomopsis*) belongs to the phylum Ascomycota and presents asexual stages, leading to difficulty in identifying members of this genus at the species level. Numerous studies and reports on the species in this genus, which has enormous biotechnological potential, have been published^28^. It has already been described as a producer of enzymes and secondary metabolites^29^ with antibacterial^30,31^ and antifungal^32,33^ properties as well as in the phytotoxicity for weeds control^34–37^. According to relevant reports, certain chemical compounds have been isolated and identified from this genus, such as camptothecin^38^ and colchicine^39^. Some of the compounds produced showed herbicidal activity. For instance, phomentrioloxin B induces little necrotic spots on several plant species, and gulypyrone A causes a leaf necrosis symptom on *Helianthus annuus*^40^. In addition, Almeida, et al.^28^ investigated the toxicity of the solid bioherbicide formulation developed by *Diaporthe* sp. on test plants of lettuce (*Lactuca sativa*).

### Comparison of the allelopathic effects of different solvent extracts of *Diaporthe*

The powdered mycelia of *Diaporthe* were weighed and subjected to solvent sequential extraction. Hexane extracted the lowest mass at 0.64 g (1.30%) and ethanol extracted the highest mass at 3.38 g (6.84%), followed by ethyl acetate at 0.94 g (1.90%). The germination percentage decreased with increasing concentrations of the extract (Table 4). Amaranth germination was inhibited by the EtOAc fraction to a greater degree than other solvents. For the initial seedling growth, all the fractions significantly reduced seedling growth except the EtOH fraction at 0.187 mg/mL. However, the root length of the weed seedling was greatly reduced than the shoot length. Hence, the EtOAc fraction had the strongest inhibitory activity.

**Table 4:** The inhibitory effects of sequential solvent extraction of *Diaporthe* sp. mycelia exert allelopathic effects on seed germination and growth of *A. tricolor*.

Solvent (concentration; mg/mL)	Inhibition level (% over control)		
	Seed germination	Shoot length	Root length
Hexane fraction			
0.750 mg/mL	8.75 cd	18.54 bc	28.95 de
0.375 mg/mL	6.25 de	15.58bcd	18.16 ef
0.187 mg/mL	1.25 e	13.51 cd	10.41 f
EtOAc fraction			
0.750 mg/mL	68.75 a	40.53 a	71.37 a
0.375 mg/mL	45.00 b	27.51 b	45.98 b
0.187 mg/mL	15.00 c	16.57 bcd	38.22 bcd
EtOH fraction			
0.750 mg/mL	8.75 cd	16.86 bcd	47.95 b
0.375 mg/mL	2.50 de	5.47 de	43.86 bc
0.187 mg/mL	1.25 e	-4.04 e	32.68 cd

The data are presented as mean. Different letters in the same column indicated significant differences according to Tukey’s multiple range tests at *p* < 0.05 level.

Important indications of plant toxicity are changes in growth and development^41^. The findings from our investigation showed that during extract treatment, the root length of *A. tricolor* seeds dramatically decreased. The roots, as the foremost organ that directly encounters the rhizosphere compound, usually accumulate more substances than the shoots^42,43^. This agrees with the findings presented by Akbar and Javaid^44^, who observed that the roots of *Rumex dentatus* were more susceptible to *Drechslera* sp. culture filtrate than were the shoots. Previous literature reveals that the possible reason for the inhibited germinated and seedling growth could be osmotic stress or an interfering enzyme during the germination process. Thus, the physiological changes of the tested seeds were further examined.

### Effect on seed imbibition and the regulation of osmolytes during seed germination

**Figure 2:**
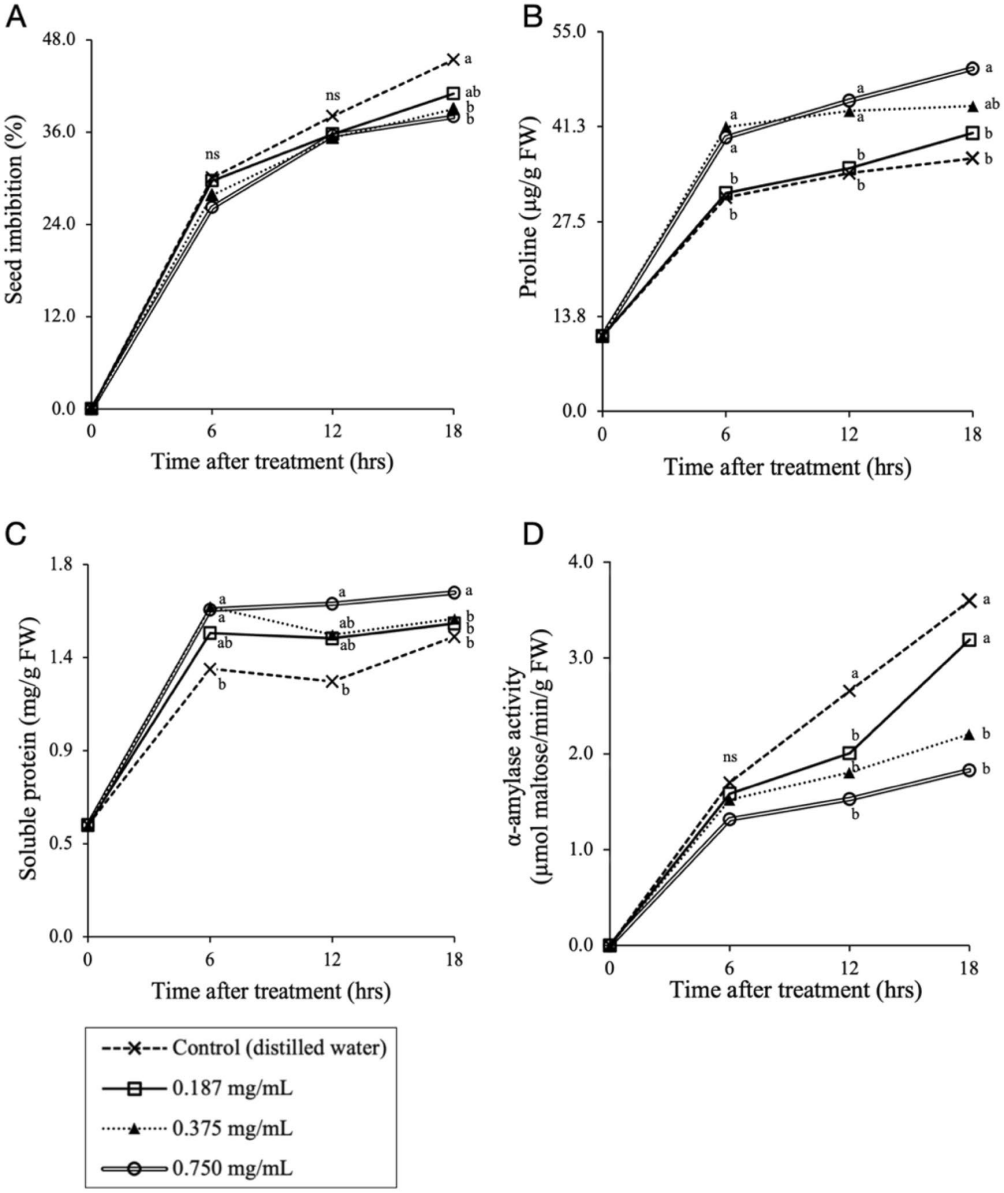
Effect of the *Diaporthe* EtOAc fraction on imbibition (**A**), proline, (**B**) soluble protein concentration (**C**), and α-amylase activity (**D**) in *A. tricolor* seeds during the germination process. The different letters in the graphs indicate significant differences (*p* < 0.05) between the treatments at the same germination time (hrs) based on Tukey’s significant difference test.

Seed imbibition, as the first step of the seed germination process, was studied. The extract was generally a weak inhibitor of the water absorption process during seed germination (Fig. 2A). The results showed that imbibition was divided into three phases: in the first 6 h after the amaranth seeds were immersed in the extract or dH_2_O, the absorbing rate of the seeds was high and was not significantly different (*p* > 0.05) than the control. In phase 2, from 6 to 12 h, the seeds absorbed water more than in phase 1, but the absorbing rate was much lower than in the first phase. However, the *Diaporthe* EtOAc fraction showed disturbed seed imbibition after 12 h of treatment (Fig. 2A). The accumulation of osmolytes is a self-production ability for maintaining the osmotic balance of plants exposed to abiotic stress^45,46^. The anti-osmotic stress regulator proline showed a slightly increasing trend across the germination time (Fig. 2B). When exposed to 0.75 mg/mL of the extract, the proline concentration in tested seeds significantly (*p* < 0.05) increased by 30.57–35.98% compared to the untreated seeds. The *Diaporthe* EtOAc fraction treatment also caused an increase in soluble protein (Fig. 2C). Compared to the control treatment, 30.56% more protein was recorded for the 0.75 mg/mL treated seed after 18 h of germination. The maximum increase was also observed with this treatment (1.66 ± 0.12 mg/g FW).

The results of our study showed that the treated seeds exhibited significantly higher levels of osmolytes, indicating that the plant cells may enhance the adaptation strategy of plant stress-resistance mechanisms^42^. Namely, the extract caused osmotic stress during seed germination. This situation limits water and mineral nutrient uptake, thereby restricting seedling growth and development, which is supported by previous studies^43,47,48^. However, the accumulated osmolytes, which struggle to obtain a normal osmotic balance stage, were insufficient to effectively ameliorate the adverse effect of the extract, as evident by the delayed imbibition (Fig. 2A) under the assay conditions. Our results supported the hypothesis that *Diaporthe* EtOAc fraction extract induced osmotic stress in *A. tricolor* seeds, which interfered with seed germination.

### Effect on α-amylase activity

Α-amylase (EC 3.2.1.1) is a major enzyme involved in germination. Upon exposure to the extract at each stage, there was a significant (*p* < 0.05) decrease in the dose-dependent response in the α-amylase activity of the seeds (Fig. 2D). Amylase activity is a crucial factor in the regulation of germination because it is an essential enzyme for the hydrolysis of the endosperm to metabolizable sugars and the provision of energy for plants throughout the seed germination process^49^. Our results showed that *Diaporthe* extract treatment significantly suppressed the α-amylase activity of the seeds. These results are in agreement with previous reports, which indicated that the inhibition of α-amylase activity by an *Alternaria brassicicola* cultured filtrate resulted in seed germination reduction^43^. These results suggest that reduced α-amylase activity may be involved in the *Diaporthe* EtOAc fraction-induced inhibition of seed germination in amaranth.

### Effect on onion root meristematic cell mitosis

**Figure 3:**
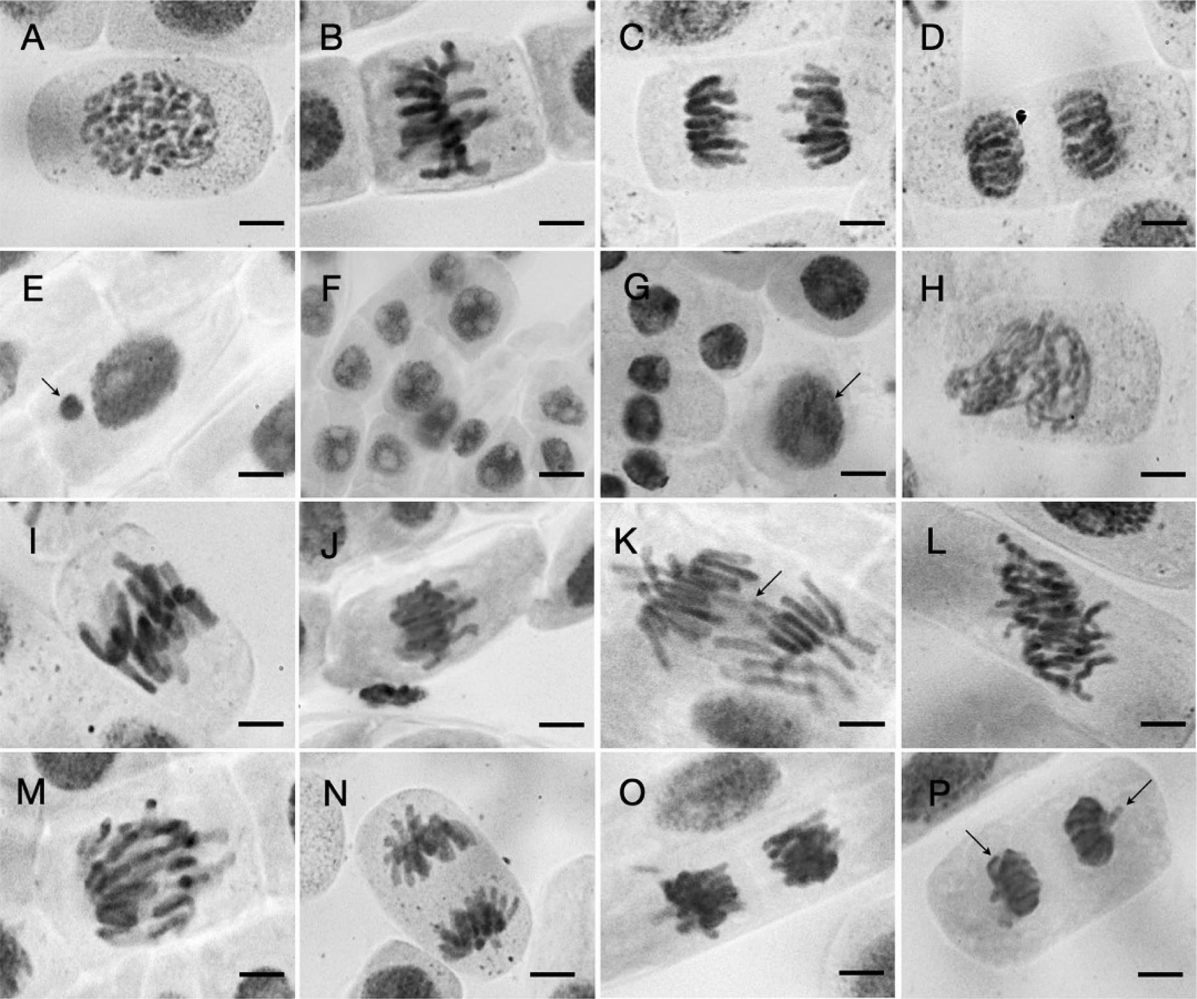
Mitotic abnormalities induced by the *Diaporthe* EtOAc fraction in the root tip cells of *A. cepa* after 12 h (**A**–**D**) typical stages of cell division; (**E**) micronucleus; (**F**) binucleated cells; (**G**) normal (arrow) and condensed nucleus; (**H**) spindle disturbance; (**I**) C-mitosis; (**J**) sticky metaphase; (**K**) anaphase bridge; (**L**) diagonal at anaphase; (**M**) delayed anaphase; (**N**,**O**) sticky anaphase; (**P**) precocious chromosomes. The bar represents 10 µm.

Mitotic observations from root meristematic cells of *A. cepa* roots demonstrated mitotic depression and a change in the proportion of the mitotic phase index (Table 5). The cell numbers in prophase (Fig. 3A), metaphase (Fig. 3B), anaphase (Fig. 3C), and telophase (Fig. 3D) were also counted as dividing stages. After incubation in the EtOAc fraction, the prophase percentage increased while the other phases decreased. Table 6 lists the types and percentages of mitotic abnormalities, and Fig. 3E–P show the associated micrographs. The *Diaporthe* EtOAc fraction induced chromosome and cytogenetic alterations in *A. cepa*. The occurrences of binucleated cells (Fig. 3F), a condensed nucleus (Fig. 3G), sticky metaphase (Fig. 3J), and an anaphase bridge (Fig. 3K) were observed at considerable frequencies with the EtOAc fraction.

**Table 5:** Mitotic index and mitotic phases of *Allium cepa* L. root tips exposed to different concentrations of *Diaporthe* sp. EtOAc extract for 12 h.

Treatments	Number of cells		Mitotic index (%)	Mitotic phase index (%)		
	Counted cells	Dividing cells		Prophase	Metaphase	Anaphase–Telophase
Control	2203	116	5.28 a	46.42 b	32.16 a	21.42 a
Surfactant	2129	112	5.09 ab	53.72 ab	28.01 a	18.27 b
Diaporthe sp. extract						
0.0625 mg/mL	2121	76	3.57 ab	49.06 ab	31.92 a	19.02 ab
0.125 mg/mL	2092	69	3.23 b	61.29 ab	26.89 a	11.82 c
0.250 mg/mL	2105	74	3.51 ab	68.74 a	22.96 a	8.30 c

Mean within columns for each *Diaporthe* sp. EtOAc extract concentration followed by different letters (a–b) is significantly different from Tukey’s multiple range tests at *p* < 0.05 level.

**Table 6:** Chromosomal aberrations in *A. cepa* root meristematic cells exposed to EtOAc fraction from *Diaporthe* sp. for 12 h.

Treatments	Chromosomal aberrations (%)									Total abnormalities (%)
	Micronucleus	Binucleated cells	Condensed nucleus	Spindle disturbance	Sticky metaphase	C-mitosis	Anaphase bridge	Sticky anaphase	Delayed anaphase	
Control	–	–	–	–	–	–	–	–	–	0.00 b
Surfactant	–	0.05	0.09	–	0.05	–	0.05	–	–	0.23 b
Diaporthe sp. extract										
0.0625 mg/mL	0.09	–	0.42	0.09	0.24	0.09	0.24	0.33	0.05	1.53 a
0.125 mg/mL	0.05	0.72	0.24	0.05	0.19	0.10	0.14	–	0.05	1.53 a
0.250 mg/mL	0.19	0.19	0.19	0.19	–	0.05	0.10	0.05	–	0.95 ab

Mean within column followed by different letters (a-b) is significantly different to Tukey’s multiple range tests at *p* < 0.05 level.

The root meristem cells of *A. cepa* were tested for cytogenetic effects. This plant is regarded as one of the most appropriate biological models for examining cell cycle disruptions because of its suitable chromosomal features^50^. The mitotic index, which is equal to the number of cells in mitotic phases, can be used as an indicator of cell growth. The mitotic index reduction observed here was a direct effect of the EtOAc fraction (Table 5). The results of mitotic activity frequencies demonstrated that the EtOAc fraction had a substantial mitodepressive impact. The reduction of mitotic activity may be related to the inhibition of DNA synthesis and/or nucleoprotein synthesis in the cell cycle^51^. In addition, our data demonstrated a change in the frequency of the mitotic phases (Table 5). The accumulation of dividing cells at prophase in this study suggested that the EtOAc fraction disrupts the processes of mitotic division and reduces the number of cells entering mitotic division, which is supported by previous publications^47,52^. Binucleated cells (Fig. 3F) and condensed nuclei (Fig. 3G), which are characterized by changes in the normal nuclei structure, also suggest a decrease in root elongation after treatment. According to Andrade-Vieira, et al.^53^, condensed nuclei are related to the programmed cell death that occurs in response to abiotic stress. The presence of micronucleated cells (Fig. 3E) was another abnormality observed in the nuclei. This nuclear formation is linked to laggards and acentric chromosomes that fail to combine into the daughter nuclei during telophase^50,54^. These occurrences are caused by spindle poisons or severe spindle damage, both of which are known to stop the progression of the cell cycle^55^. As a consequence of the toxic effect on the destruction or incomplete formation of the mitotic spindle, spindle disturbances occurred at late prophase (Fig. 3H), delayed anaphase (Fig. 3M), the anaphase bridge (Fig. 3K), and diagonally at the anaphase (Fig. 3L). In addition, the presence of C-mitosis (Fig. 3I) could be the result of the complete inactivation of mitotic spindles. The possible induction of aneuploidy and polyploidy at the final stages of cell division could result from a defective mitotic spindle formation at various phases of mitotic division^56^. Chromosome stickiness during metaphase (Fig. 3J) and anaphase (Fig. 3N,O) Cell growth in plants is dependent on mitotic processes, as cells undergo interphase and mitosis phases to complete the cell cycle. Most seedlings die before emerging when cell division is halted during seed germination.

## Materials and methods

### Surface sterilization and isolation of phytopathogenic fungi

Weeds with some infection symptoms were obtained from the Ladkrabang district of Bangkok, Thailand in September 2021, for the isolation of the fungi (Table 1). The research on this plant species has complied with relevant institutional, national, and international guidelines and legislation. The collected samples were subjected to surface sterilization procedures and the tissue transplanting method in Department of Plant Production Technology Laboratory, School of Agricultural Technology, KMITL. After sterilization, the diseased tissues were further cut from the advanced margin of the lesions to expose the interior surface to water agar (WA). The Petri dishes were sealed with parafilm and incubated at 27 °C for 7 days. Subcultures from the master plates were made by transferring the hyphal tips to fresh potato dextrose agar (PDA) plates and incubating them to obtain pure cultures. The purified fungal isolates were then transferred to PDA slants and maintained at 4 °C until further use.

### Fermentation and extraction of fungi

All the isolated fungi were codified and grown in the submerged fermentation process (SMF) in the first step. The fungal discs of the isolated fungal strain were transferred to the fermentation Petri dishes (90 mm × 15 mm) containing autoclaved fermentation broth under aseptic conditions. The fermentation broth was composed of 50.0 g/L potato extract and 20.0 g/L glucose, and the final pH was adjusted to 5.0 ± 0.2. The inoculated Petri dishes were incubated at room temperature for 30 days. After incubation, each fungal mat was collected from the medium through a sterilized muslin cloth and then incubated at 45 °C in a hot air oven (Binder World FP400UL-208 V, Binder, Germany) for 48 h to air-dry. The dried mycelia were ground and extracted (ratio 1.0 g of dried mycelia: 20.0 mL of solvents) with absolute ethanol (EtOH) containing 25% volume of dH_2_O (75% EtOH) at room temperature. After 7 days of extraction, the supernatants were filtered with cotton and re-filtered through Whatman No. 1 filter paper (Whatman Inc., Clifton, USA). The resulting filtrate solutions were evaporated using a rotary vacuum evaporator (BUCHI Rotavapor R255, BUCHI, Lausanne, Switzerland) under partial vacuum at 45 °C to obtain the crude extract. When a consistent yield of crude was acquired, the extracts were air-dried and weighed. Then, each crude extract was stored at 4 °C for further experiments.

### *Selection of potential fungi *via* a seed germination bioassay*

*A. tricolor* seeds were purchased from Chia Tai Co. Ltd., Bangkok, Thailand. Each sticky fungal crude extract was formulated first by preparing an anionic surfactant mixture, including Tween® 80: dimethyl sulfoxide (DMSO); 4:3 (v/v). Then, the crude extract was dissolved at a 3:7 ratio (fungal crude extract: surfactant mixture; w/v). The components of each formula were homogenized using a mechanical stirrer. After homogenization, the liquid solution was used as a stock solution. Each fungal extract stock solution was dissolved in dH_2_O to a concentration of 1.50 mg/mL and was directly used in the bioassays. Seed germination tests were done by adding 5.0 mL of formulant to the germination paper and placing each in a glass Petri dish. Then, we selected full-particle and equal-sized *A. tricolor* seeds and placed them in Petri dishes (20 seeds per dish). The treatment with a surfactant mixture and dH_2_O served as a check and control. The germination of the tested seeds, root, and shoot length (cm) was recorded after 7 days of treatment. The experiment was done using a completely randomized design (CRD) with four replicates. The inhibition percentage was then determined. The two fungal crude extracts that presented the highest inhibition were diluted to a lower concentration to study the growth response. After dilution, the Petri dish seed germination assay was conducted as described above.

### Molecular identification of the herbicidal strains

The strain with herbicidal activity was identified by DNA sequencing methods. DNA was extracted from the pure culture. The fungal genomic DNA was isolated using the CTAB method^57^. The internal transcribed spacer (ITS) region of fungal ribosomal DNA (rDNA) was amplified with the specific universal primers ITS1 (5’ − TCCGTAGGTGAACCTTGCGG − 3’) and ITS4 (5’ − TCCTCCGCTTATTGATATGC − 3’). The polymerase chain reaction (PCR) amplification was performed using a thermal cycler (BioRad®, USA). The PCR reaction conditions were set at 95 °C for 2 min, followed by 35 cycles of denaturation at 94 °C for 30 s, annealing at 52 °C for 30 s, and extension at 72 °C for 90 s, with a final extension at 72 °C for 15 min. The PCR amplicons were sent to be purified and sequenced at Marcogen™ (Seoul, Korea). For the identification, the obtained sequences were compared using a similarity index between sequences with the available sequences in the NCBI (National Center for Biotechnology Information) database using the BLASTn search program. The sequences were edited using BioEdit 7.2 software. The newly obtained sequences were aligned with highly similar, homologous sequences from the NCBI nucleotide database, which confirmed the species-level similarity with the query sequence of the isolates. The ITS rRNA gene sequences were aligned using the ClustalW algorithm with default parameters for the phylogenetic analysis. A Maximum Likelihood tree method was constructed based on the analysis of ITS regions. The percentage of replicate trees in which the taxa clustered together in the bootstrap test (1,000 replicates) is shown next to the branches. Evolutionary analyses were conducted using MEGA 11 software.

### *Extraction of the herbicidal compounds *via* sequential extraction*

The *Diaporthe* strain was cultured in the SMF for 30 days as described above. The collected mycelium powder (MY, 49.40 g) was extracted with three different organic solvents by increasing the polarity with hexane (Hex), ethyl acetate (EtOAc), and absolute ethanol (EtOH). After 24 h of extraction, the mycelium extract fractions were ultrasonically oscillated for 30 min at 50 °C. The supernatants were filtered through Whatman paper. Following filtration, the extracts were evaporated under a partial vacuum to obtain a crude fraction. Each fraction obtained was dissolved with its extracted solvent, and the concentrations were adjusted to a series of 0.750–0.187 mg/mL and added to the glass Petri dish. The covers of the dishes were left open overnight to completely evaporate the solvent. Then, 5.0 mL of dH_2_O was added. The crude fractions were evaluated for herbicidal activity using the Petri dish assay as described above.

### Seed imbibition

Seed imbibition was measured at 6, 12, and 18 h after exposure to 100 *A. tricolor* seeds per treatment. Briefly, the initial seed weight (W1) was recorded, and then, the seeds were soaked in the extract solution at each concentration and exposure time. After incubation, the seeds were washed and weighed (W2). The water absorption values are expressed in seed imbibition percentages. The experiment had three replicates (300 seeds in total) per treatment, arranged in a CRD.

$$\mathrm{Seed imbibition }\left(\mathrm{\%}\right)=\frac{\text{Grams of solution absorbed} -\text{ Gram of dry seeds}}{\text{Gram of dry seeds}}\times 100$$

### Determination of osmolytes

Full-particle and equal-sized amaranth seeds were soaked with the herbicidal activity fraction solution as described in “Effect on onion root meristematic cell mitosis”. The proline concentration was estimated following the standard procedure described by Bates et al.^58^, with slight modifications. First, 0.1 g of the sample was homogenized with 4.0 mL of sulfosalicylic acid (3% w/v), followed by centrifugation (10,000 rpm) for 10 min at 4 °C. The 1.0 mL supernatant was mixed with glacial acetic acid (3.0 mL), ninhydrin (1.0 mL), and distilled water (2.0 mL) and incubated at 100 °C for 30 min. After cooling at room temperature, the mixture was added to 6.0 mL of toluene, and the absorption of the extract was measured at 520 nm. The proline concentration was determined after the realization of a standard curve and is expressed as µg/g FW. The detection of the soluble proteins was determined following the Coomassie Brilliant Blue method^59^ and calculated from a standard bovine serum albumin (BSA) curve.

### Determination of α-amylase activity

The enzymatic activity of α-amylase (EC 3.2.1.1) was determined by the 3,5-dinitrosalicylic acid (DNS) assay. The seeds were grained with 4.0 mL of ice-cold 0.1 M CaCl_2_ solution and centrifuged at 10,000 rpm for 20 min at 4 °C. The supernatant was collected and used as an enzyme source. The reaction was done by mixing 1.0 mL of the enzyme with 1.0 mL of 0.5% w/v soluble starch as the substrate. The mixture was incubated, and 1.0 mL of DNS reagent was added to the mixture. Finally, absorption at 560 nm was measured.

### Cytogenetic assay

As the study material, equal-sized bulbs of onion (*Allium cepa* L.; 2n = 16) were used for the cytogenetic experiments. The emerged onion roots were treated with a series of concentrations of the EtOAc fraction (0.0625, 0.125, and 0.250 mg/mL) for 12 h. After the end of the exposure period, root tips were collected and subsequently fixed in a freshly prepared fixative solution. Then, the fixed root tips were washed in dH_2_O and hydrolyzed in an enzyme mixture containing 8% w/v cellulase and 6% w/v pectinase in a 0.01 M acetate buffer (pH 4.5) for 40 min at 37 °C. To prepare the slides, the meristematic region was squashed onto a drop of 2% Giemsa solution (Merck Co., Ltd.). The following parameters were calculated: mitotic index, mitotic phase index, and chromosome aberrations according to the methods described by Aragão, et al.^50^.

## Conclusions

During this screening process, all the isolates were isolated and subsequently assessed for their phytotoxic impact in *A. tricolor*. The fungus designated as EC010 exhibited the most beneficial results and was classified as belonging to the genus *Daiporthe*. The inhibitory effects of the EtOAc fraction on seed germination may be attributed to its impact on imbibition, α-amylase activity, and cell division, leading to a decrease in these processes. Hence, it is possible that this extract possesses the capacity to be further refined and developed into a viable natural herbicide in the future.

## Data Availability

The *Diaporthe* sp. EC-010 sequence data are available in the NCBI repository (https://www.ncbi.nlm.nih.gov/), accession number: OR143425.

## References

[1] Sharma A (2020). Global trends in pesticides: A looming threat and viable alternatives. Ecotoxicol. Environ. Saf..

[2] Damalas CA, Eleftherohorinos IG (2011). Pesticide exposure, safety issues, and risk assessment indicators. Int. J. Environ. Res. Public Health.

[3] Brookes G (2019). Glyphosate use in Asia and implications of possible restrictions on its use. AgBioforum.

[4] Walsh A, Kingwell R (2021). Economic implications of the loss of glyphosate and paraquat on Australian mixed enterprise farms. Agric. Syst..

[5] Kim J-W, Kim D-S (2020). Paraquat: toxicology and impacts of its ban on human health and agriculture. Weed Sci..

[6] Camargo ER (2020). Current situation regarding herbicide regulation and public perception in South America. Weed Sci..

[7] Cancer, I. A. F. R. O. *IARC Monographs Volume 112: Evaluation of Five Organophosphate Insecticides and Herbicides* (World Health Organization, 2015).

[8] Andreotti G (2018). Glyphosate use and cancer incidence in the agricultural health study. JNCI J. Natl. Cancer Inst..

[9] Beckie HJ, Flower KC, Ashworth MB (2020). Farming without Glyphosate?. Plants.

[10] Hoagland RE, Boyette CD, Weaver MA, Abbas HK (2007). Bioherbicides: Research and risks. Toxin Rev..

[11] Bailey KL, Pitt W, Leggett F, Sheedy C, Derby J (2011). Determining the infection process of *Phoma*
*macrostoma* that leads to bioherbicidal activity on broadleaved weeds. Biol. Control.

[12] Cordeau S, Triolet M, Wayman S, Steinberg C, Guillemin J-P (2016). Bioherbicides: Dead in the water? A review of the existing products for integrated weed management. Crop Prot..

[13] Souza ARCD (2017). Selection, isolation, and identification of fungi for bioherbicide production. Braz. J. Microbiol..

[14] Latif, S., Chiapusio, G. & Weston, L. *Advances in Botanical research* Vol. 82 19–54 (Elsevier, 2017).

[15] Motlagh MRS (2012). Evaluation of *Alternaria*
*alternata* causing leaf spot of barnyardgrass grown in rice fields. Afr. J. Microbiol. Res..

[16] Jiang SJ, Qiang S, Zhu YZ, Dong YF (2008). Isolation and phytotoxicity of a metabolite from *Curvularia*
*eragrostidis* and characterisation of its modes of action. Ann. Appl. Biol..

[17] Tessmann DJ, Charudattan R, Preston JF (2008). Variability in aggressiveness, cultural characteristics, cercosporin production and fatty acid profile of *Cercospora*
*piaropi*, a biocontrol agent of water hyacinth. Plant Pathol..

[18] Bowling AJ, Vaughn KC, Hoagland RE, Stetina K, Boyette CD (2010). Immunohistochemical investigation of the necrotrophic phase of the fungus Colletotrichum gloeosporioides in the biocontrol of hemp sesbania (*Sesbania*
*exaltata*; Papilionaceae). Am. J. Bot..

[19] Piyaboon O, Pawongrat R, Unartngam J, Chinawong S, Unartngam A (2016). Pathogenicity, host range and activities of a secondary metabolite and enzyme from *Myrothecium*
*roridum* on water hyacinth from Thailand. Weed Biol. Manag..

[20] Hubbard M, Hynes R, Bailey K (2015). Impact of macrocidins, produced by Phoma macrostoma, on carotenoid profiles of plants. Biol. Control.

[21] Héraux FM, Hallett SG, Weller SC (2005). Combining Trichoderma virens-inoculated compost and a rye cover crop for weed control in transplanted vegetables. Biol. Control.

[22] Radhakrishnan R, Alqarawi AA, Abd Allah EF (2018). Bioherbicides: Current knowledge on weed control mechanism. Ecotoxicol. Environ. Saf..

[23] Venkatasubbaiah P, Baudoin A, Chilton W (1992). Leaf spot of hemp dogbane caused by *Stagonospora*
*apocyni*, and its phytotoxins. J. Phytopathol..

[24] Varkonda, S. Herbicides of microbial origin [Bialaphos, Anisomycin, Metoxyphenone, Cycloheximide, Streptomyces spp.]. *Agrochemia (Czechoslovakia)* (1985).

[25] Zhang LH, Kang ZH, Jiao X, Xu WC, Zhang JL (2010). Isolation and structural indentification of herbicidal toxin fractions produced by *Pythium*
*aphanidermatum*. Agric. Sci. China.

[26] Rebordinos L, Cantoral JM, Prieto MV, Hanson JR, Collado IG (1996). The phytotoxic activity of some metabolites of *Botrytis*
*cinerea*. Phytochemistry.

[27] Santos GD (2021). Molecular identification and antimicrobial activity of foliar endophytic fungi on the Brazilian pepper tree (*Schinus*
*terebinthifolius*) reveal new species of *Diaporthe*. Curr. Microbiol..

[28] Almeida TC (2020). Development of a solid bioherbicide formulation by spray drying technology. Agriculture.

[29] Chepkirui C, Stadler M (2017). The genus Diaporthe: A rich source of diverse and bioactive metabolites. Mycol. Progress.

[30] Medeiros AG (2018). Bioprospecting of *Diaporthe*
*terebinthifolii* LGMF907 for antimicrobial compounds. Folia Microbiol..

[31] Lin X (2005). Cytotoxic and antimicrobial metabolites from marine lignicolous fungi *Diaporthe* sp. FEMS Microbiol. Lett..

[32] Reis CM (2019). Antifungal and antibacterial activity of extracts produced from *Diaporthe*
*schini*. J. Biotechnol..

[33] Tanney JB, McMullin DR, Green BD, Miller JD, Seifert KA (2016). Production of antifungal and antiinsectan metabolites by the Picea endophyte *Diaporthe*
*maritima* sp. nov. Fungal Biol..

[34] Souza ARC (2015). Bioherbicide production by *Diaporthe* sp. isolated from the Brazilian Pampa biome. Biocatal. Agric. Biotechnol..

[35] Pes MP (2016). Bioherbicide based on *Diaporthe* sp. secondary metabolites in the control of three tough weeds. Afr. J. Agric. Res..

[36] Bastos BDO (2017). Solid-state fermentation for production of a bioherbicide from *Diaporthe* sp. and its formulation to enhance the efficacy. 3 Biotech.

[37] Brun T (2022). Weed control by metabolites produced from *Diaporthe*
*schini*. Environ. Technol..

[38] Degambada K, Kumara P, Salim N, Abeysekera A, Chandrika U (2023). Diaporthe sp F18; A new source of camptothecin-producing endophytic fungus from *Nothapodytes*
*nimmoniana* growing in Sri Lanka. Nat. Prod. Res..

[39] Deepika VB (2020). DNA demethylation overcomes attenuation of colchicine biosynthesis in an endophytic fungus Diaporthe. J. Biotechnol..

[40] Andolfi A (2015). Gulypyrones A and B and Phomentrioloxins B and C produced by Diaporthe gulyae, a potential mycoherbicide for saffron thistle (*Carthamus*
*lanatus*). J. Nat. Prod..

[41] Mimouni H (2016). Does salicylic acid (SA) improve tolerance to salt stress in plants? A study of SA effects on tomato plant growth, water dynamics, photosynthesis, and biochemical parameters. Omics.

[42] Kamran M (2021). Pre-sowing seed treatment with kinetin and calcium mitigates salt induced inhibition of seed germination and seedling growth of choysum (*Brassica*
*rapa* var. parachinensis). Ecotoxicol. Environ. Saf..

[43] Manichart N, Somala N, Laosinwattana C (2023). Allelopathic potential of secondary metabolites produced by *Alternaria **brassicicola* and physiological mechanisms on *Amaranthus **tricolor*. Int. J. Agric. Technol..

[44] Akbar, M. & Javaid, A. Management of some problematic weeds of wheat by metabolites of *Drechslera* sp. prepared in malt extract medium. *Pak. J. Weed Sci. Res.* **16** (2010).

[45] Kamran M (2020). Modulation of growth performance and coordinated induction of ascorbate-glutathione and methylglyoxal detoxification systems by salicylic acid mitigates salt toxicity in choysum (*Brassica*
*parachinensis* L.). Ecotoxicol. Environ. Saf..

[46] Khalil R, Haroun S, Bassyoini F, Nagah A, Yusuf M (2021). Salicylic acid in combination with kinetin or calcium ameliorates heavy metal stress in *Phaseolus*
*vulgaris* plant. J. Agric. Food Res..

[47] Teerarak M, Charoenying P, Laosinwattana C (2012). Physiological and cellular mechanisms of natural herbicide resource from *Aglaia*
*odorata* Lour. on bioassay plants. Acta Physiol. Plant..

[48] Laosinwattana C, Wichittrakarn P, Teerarak M (2018). Chemical composition and herbicidal action of essential oil from *Tagetes*
*erecta* L. leaves. Ind. Crops Prod..

[49] Li Q, Yang A, Zhang W-H (2019). Higher endogenous bioactive gibberellins and α-amylase activity confer greater tolerance of rice seed germination to saline-alkaline stress. Environ. Exp. Bot..

[50] Aragão F (2015). Phytotoxic and cytotoxic effects of Eucalyptus essential oil on lettuce (*Lactuca*
*sativa* L.). Allelopathy J.

[51] Türkoğlu Ş (2012). Determination of genotoxic effects of chlorfenvinphos and fenbuconazole in Allium cepa root cells by mitotic activity, chromosome aberration, DNA content, and comet assay. Pesticide Biochem. Physiol..

[52] Charoenying P, Teerarak M, Laosinwattana C (2010). An allelopathic substance isolated from Zanthoxylum limonella Alston fruit. Sci. Hortic..

[53] Andrade-Vieira L, Gedraite L, Campos J, Davide L (2011). Spent Pot Liner (SPL) induced DNA damage and nuclear alterations in root tip cells of Allium cepa as a consequence of programmed cell death. Ecotoxicol. Environ. Saf..

[54] Yi H, Meng Z (2003). Genotoxicity of hydrated sulfur dioxide on root tips of Allium sativum and Vicia faba. Mutat. Res./Genet. Toxicol. Environ. Mutagen..

[55] Joglekar AP (2016). A cell biological perspective on past, present and future investigations of the spindle assembly checkpoint. Biology.

[56] Wang G-F (2017). Oxidative stress induces mitotic arrest by inhibiting Aurora A-involved mitotic spindle formation. Free Radic. Biol. Med..

[57] White TJ, Bruns T, Lee S, Taylor J (1990). Amplification and direct sequencing of fungal ribosomal RNA genes for phylogenetics. PCR Protocols.

[58] Bates LS, Waldren RP, Teare ID (1973). Rapid determination of free proline for water-stress studies. Plant Soil.

[59] Chen L (2020). Exogenous melatonin promotes seed germination and osmotic regulation under salt stress in cotton (*Gossypium*
*hirsutum* L.). PLoS ONE.

